# Concurrent definitive chemoradiation incorporating intensity-modulated radiotherapy followed by adjuvant chemotherapy in high risk locally advanced cervical squamous cancer: a phase II study

**DOI:** 10.1186/s12885-022-10406-9

**Published:** 2022-12-20

**Authors:** Gong-yi Zhang, Rong Zhang, Ping Bai, Shu-min Li, Yuan-yuan Zhang, Yi-ran Chen, Man-ni Huang, Ling-ying Wu

**Affiliations:** Department of Gynecological Oncology, National Cancer Center/National Clinical Research Center for Cancer/Cancer Hospital, Chinese Academy of Medical Sciences and Peking Union Medical College, NO.17 Panjiayuan, Chaoyang District, Beijing, 100021 China

**Keywords:** Cervical cancer, IMRT, Adjuvant chemotherapy

## Abstract

**Background:**

Although the prognosis of locally advanced cervical cancer has improved dramatically, survival for those with stage IIIB-IVA disease or lymph nodes metastasis remains poor. It is believed that the incorporation of intensity-modulated radiotherapy into the treatment of cervical cancer might yield an improved loco-regional control, whereas more cycles of more potent chemotherapy after the completion of concurrent chemotherapy was associated with a diminished distant metastasis. We therefore initiated a non-randomized prospective phaseII study to evaluate the feasibility of incorporating both these two treatment modality into the treatment of high risk locally advanced cervical cancer.

**Objectives:**

To determine whether the incorporation of intensity-modulated radiotherapy and the addition of adjuvant paclitaxel plus cisplatin regimen into the treatment policy for patients with high risk locally advanced cervical cancer might improve their oncologic outcomes.

**Study design:**

Patients were enrolled if they had biopsy proven stage IIIA-IVA squamous cervical cancer or stage IIB disease with metastatic regional nodes. Intensity-modulated radiotherapy was delivered with dynamic multi-leaf collimators using 6MV photon beams. Prescription for PTV ranged from 45.0 ~ 50.0 Gy at 1.8 Gy ~ 2.0 Gy/fraction in 25 fractions. Enlarged nodes were contoured separately and PTV-nodes were boosted simultaneously to a total dose of 50.0–65 Gy at 2.0- 2.6 Gy/fraction in 25 fractions. A total dose of 28 ~ 35 Gy high-dose- rate brachytherapy was prescribed to point A in 4 ~ 5 weekly fractions using an iridium- 192 source. Concurrent weekly intravenous cisplatin at 30 mg/m^2^ was initiated on the first day of radiotherapy for over 1-h during external-beam radiotherapy. Adjuvant chemotherapy was scheduled within 4 weeks after the completion of concurrent chemo-radiotherapy and repeated 3 weeks later. Paclitaxel 150 mg/m^2^ was given as a 3-h infusion on day1, followed by cisplatin 35 mg/m^2^ with 1-h infusion on day1-2 (70 mg/m^2^ in total).

**Results:**

Fifty patients achieved complete response 4 weeks after the completion of the treatment protocol, whereas 2 patients had persistent disease. After a median follow-up period of 66 months, loco-regional (including 2 persistent disease), distant, and synchronous treatment failure occurred in 4,5, and 1, respectively. The 5-year disease-free survival, loco-regional recurrence-free survival, distant-metastasis recurrence-free survival was 80.5%, 90.3%, and 88.0%, respectively. Four of the patients died of the disease, and the 5-year overall survival was 92.1%. Most of the toxicities reported during concurrent chemo-radiotherapy were mild and transient. The occurrence of hematological toxicities elevated mildly during adjuvant chemotherapy, as 32% (16/50) and 4% (2/50) patients experienced grade 3–4 leukopenia and thrombocytopenia, respectively. Grade 3–4 late toxicities were reported in 3 patients.

**Conclusions:**

The incorporation of intensity-modulated radiotherapy and adjuvant paclitaxel plus cisplatin chemotherapy were highly effective and well-tolerated in the treatment of high-risk locally advanced cervical cancer. The former yields an improved loco-regional control, whereas distant metastases could be effectively eradicated with mild toxicities when adjuvant regimen was prescribed.

## Highlights

1. Incorporating IMRT and adjuvant chemotherapy into the treatment of locally advanced cervical cancer is highly effective.

2. IMRT is associated with a reduced loco-regional relapse.

3. Adding adjuvant TP chemotherapy could diminish tumor hematological spread.

4. Toxicity profiles were mild and manageable.

## Introduction

Although the prognosis of locally advanced cervical cancer (LACC) has improved dramatically, survival for those with stage IIIB-IVA disease or lymph nodes metastasis (LNM) remains poor. If treated with conventional radiotherapy alone, approximately 46% ~ 78% stage IIIB-IVA disease would relapse at 5 years, and the outcomes for those with LNM seemed to be worse [1–5]. Even after concurrent chemoradiotherapy (CCRT) became the standard of care for LACC, nearly 50% of this subgroup of patients would develop treatment failure [6–8]. Thus, this subgroup of patients was classified as “high-risk”.

In recent decades, intensity-modulated radiotherapy (IMRT) has become the mainstream of treatment for patients with prostate, rectal, neck, and several other malignancies [9–12]. Theoretically, its incorporation into the treatment of LACC might yield an improved loco-regional control as well, as it allows for an escalated prescription dose to target volumes while sparing normal tissues from excessive radiation. However, several concerns still exist, including prolonged treatment time, geographical target miss, organ motion and set-up errors, etc. Therefore, further evidence supporting its regular use in the treatment of LACC is warranted.

Potential benefit of adjuvant chemotherapy following CCRT could firstly be implied in the reports published by Morris et al., who revealed that patients prescribed with more cycles of higher dose chemotherapy demonstrated a diminished risk of distant metastasis (DM) [13]. Nevertheless, investigations carried out thereafter were mainly phase II trials composed of heterogeneous patients treated with various cytotoxic combinations, thus conflicting and ambiguous results were usually reported [14–21].

We therefore initiated a non-randomized prospective phaseII study to investigate whether the incorporation of IMRT and adjuvant paclitaxel plus cisplatin (TP) regimen, which had been revealed to be the most, or at least one of the most active cytotoxic combinations in the treatment of advanced or recurrent cervical cancer [22], would improve the treatment outcome of high-risk LACC patients. The primary endpoints of this study were disease-free survival (DFS) and overall survival (OS). The secondary endpoints included the patterns of failure and toxicity profiles.

## Materials and methods

### Eligibility

Patients were enrolled if they had biopsy proven stage IIIA-IVA squamous cervical cancer or stage IIB disease with metastatic regional nodes. Tumor staging was defined according to the International Federation of Obstetrics and Gynecology (FIGO) system. Lymph nodes were classified as metastatic based on their radiographic findings (≥ 1.0 cm in the short-axis dimension). Eligibility criteria also included: age ≤ 70 years, Eastern Cooperative Oncology Group (ECOG) performance status (PS) ≤ 1, no previous history of chemotherapy or radiotherapy, sufficient bone marrow, adequate renal and hepatic functions. Patients with synchronous malignancies, distant metastases, known hypersensitivity to cisplatin or paclitaxel, or those with poorly controlled medical conditions would be excluded.

### Pretreatment workup

Pretreatment workup included detailed medical history, gynecological examination, complete blood count, blood chemistry, and chest X-ray. Lymph node involvement and parametrial infiltration were evaluated by magnetic resonance imaging (MRI) and/or abdomino-pelvic computer tomography (CT), and the former was preferred. If rectal or bladder invasion were suspected, additional rectoscopy or cystoscopy would be performed. Written informed consents was obtained from participants prior to the initiation of medical intervention. The study was approved by the Institutional Review Board and abided with the ethical standards of the Helsinki Declaration on good clinical practice. This study was registered with the ISRCTN registry (Registration No.: ISRCTN53385401, Date of Registration: 26/10/2021).

### Treatment schedule

**Fig. 1:**
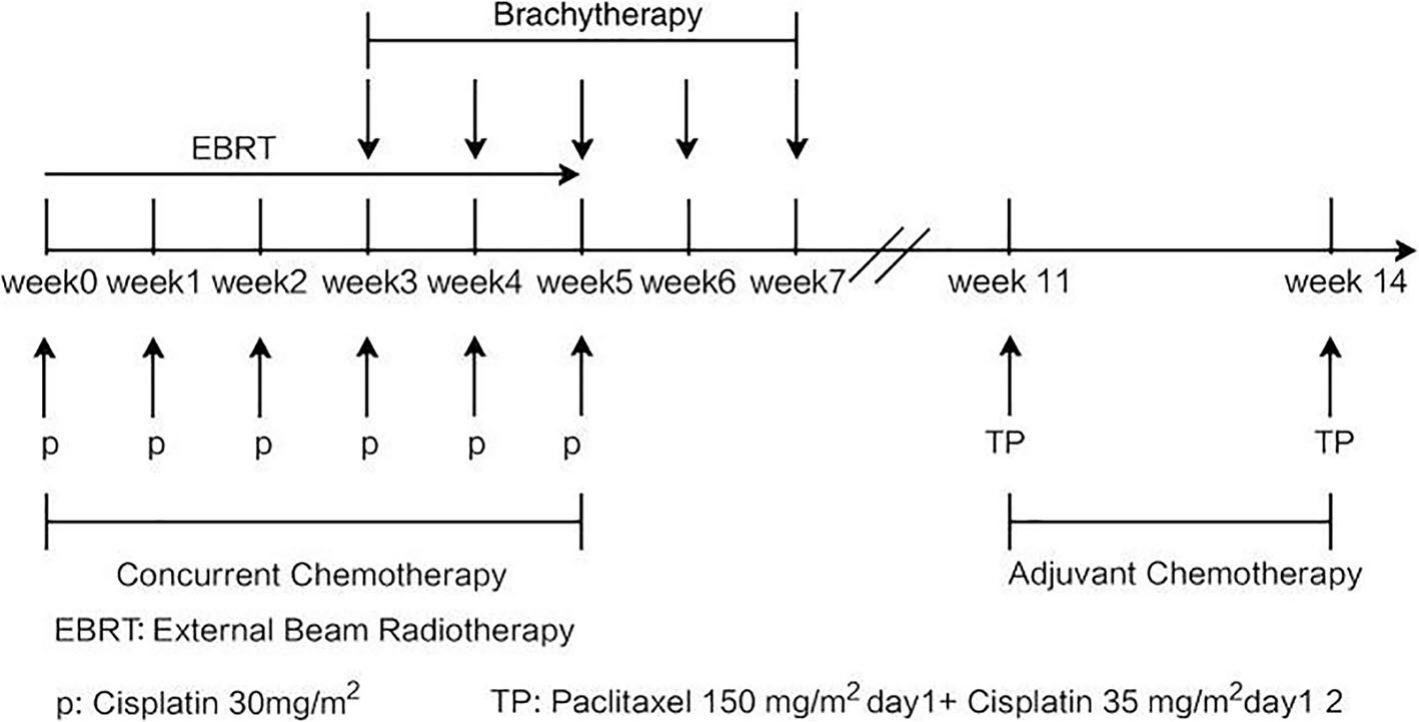
Treatment schedule for patients treated with concurrent CCRT incorporation IMRT followed by adjuvant TP regimen. EBRT: External Beam Radiotherapy p: Cisplatin 30 mg/m2 TP: Paclitaxel 150 mg/m2 d1 + Cisplatin 35 mg/m2 d1-d2

Treatment schedule was outlined in Fig. 1. IMRT was delivered with dynamic multi-leaf collimators using 6MV photon beams. A 5 mm slice thickness CT simulation was carried out in the supine position. Both oral and intravenous administrations of contrast agents were used. A comfortably full bladder and empty rectum were required. Patients were instructed to empty rectum and bladder and consume nearly 1000 ml water over 15 min, so that bladder volume would be nearly 300-400 ml at 45 min later when CT-based simulation was carried out.

Planning was performed using the Pinnacle3 9.0 treatment planning systems (Philips Healthcare, Andover, MA, USA). The Radiation Therapy Oncology Group (RTOG) consensus guidelines were followed to delineate clinical target volume (CTV) and organs at risk (OARs). CTV was defined as the gross tumor plus areas potentially containing microscopic disease, generally consisting of a 1–2 cm margin around the cervix, uterus, parametria, presacral space, lymph drainage area, and superior third of the vagina. The common iliac, external iliac, internal iliac, obturator, and lower part of paraaortic (2 cm above the aortic bifurcation) nodal volume was contoured based on the contrast-enhanced vessels with a 7 mm circumferential margin. In patients with bulky pelvic LNs (> 2 cm in shortest axis) or involved common iliac nodes or beyond, it would be contoured to the level of renal arteries (extended field-MIRT, EF-IMRT). In addition, organ motion and filling status of bladder/rectum was further accounted for during the contouring of CTV. CTV would be individualized and modified according to the organ motion visually assessed between the pretreatment imaging and planning CT with different filling of the bladder. If the rectum was distended in planning CT, additional margin would be added in the posterior direction as the rectum was supposed to be empty during treatment.

The CTV was expanded by 5 mm uniformly to create the planning target volume (PTV). Prescription for PTV ranged from 45.0 ~ 50.0 Gy at 1.8 Gy ~ 2.0 Gy/fraction in 25 fractions. Involved nodes were contoured separately and were defined as GTV-nodes. A tailored margin of 3 mm was added to GTV-nodes to generate PTV-nodes, which were treated with a simultaneous integrated boost (SIB) technique to a total dose of 50.0–65 Gy at 2.0- 2.6 Gy/fraction in 25 fractions. The IMRT plans were optimized to deliver 100% of the prescription dose to a minimum of 95% of the volume of PTV while minimizing the volume receiving 110% of the prescription dose. Dose–volume constraints for normal tissues included the following: bladder V_40_ < 50% and V_50_ < 20%, rectum V_40_ < 50% and V_50_ < 20%, intestine V_40_ < 50%, pelvic bones V_30_ < 40%, femoral heads V_50_ < 5%. Electronic portal imaging was taken for setup verification for the first 3 consecutive days.

Two-dimension high-dose- rate (HDR) brachytherapy based on orthogonal X-rays was initiated using an intrauterine tandem when 27.0 ~ 30.0 Gy of external beam was delivered to PTV. According to the guideline of National Comprehensive Cancer Network, the aim of brachytherapy boost in the current study was to deliver cumulative EQD2 doses (combined external beam radiotherapy (EBRT) and brachytherapy delivered in 2 Gy equivalent doses) of ≥ 80 Gy to point A for stageIIB-IIIA disease and ≥ 90 Gy for stage IIIB-IVA disease. Specifically, a total dose of 28 ~ 35 Gy was prescribed to point A in 4 ~ 5 weekly fractions using an iridium-192 source. An additional fraction of 5-7 Gy brachytherapy would be delivered if residual cervical tumor was suspected by pelvic examination or MRI. The doses to the bladder and the rectum were quantified using the reference points defined by the International Commission on Radiation Units and Measurements (ICRU) report 38 and were limited to < 80% of prescribed point A dose.

Chemotherapy consisted of 4 ~ 6 cycles of concurrent cisplatin infusions and 2 cycles of adjuvant TP regimen. Concurrent weekly intravenous cisplatin at 30 mg/m^2^ was initiated on the first day of radiotherapy for over 1-h during EBRT. Adjuvant chemotherapy was scheduled within 4 weeks after the completion of CCRT and repeated 3 weeks later. Paclitaxel 150 mg/m^2^ was given as a 3-h infusion on day1, followed by cisplatin 35 mg/m^2^ with 1-h infusion on day1-2 (70 mg/m^2^ in total). Discontinuation of chemotherapy was allowed in the event of grade 3–4 hematological or gastrointestinal toxicities. It would be resumed when patients’ absolute neutrophil count recovered to ≥ 1500/mm^3^ and their platelet count improved to ≥ 100,000/mm^3^; however, doses of all agents should be subsequently reduced by 20%.

### Toxicity assessment

The patients were assessed for toxicities twice per week during treatment. Complications occurred within 90 days of the initiation of chemoradiation were classified as acute complications, whereas those occurred afterwards were classified as late complications. The severity of acute complications was classified according to the Common Terminology Criteria for Adverse Events (CTCAE) v4.0. Late complications were graded according to the Radiation Therapy Oncology Group (RTOG) Late Radiation Morbidity Scoring Scheme.

### Follow up

Post-treatment response was assessed based on pelvic examination and pelvic MRI or CT 4 weeks after the completion of treatment schedule. Continued surveillance was conducted at 3-month intervals for 2 years, every 6 months during the next 3 years, and annually thereafter. At each follow-up visit, pelvic examination including Pap smear and HPV detection was routinely performed, whereas imaging including ultrasound, chest x-ray, CT, or MRI were prescribed at physician’s discretion.

Patterns of failure were analyzed in terms of loco-regional recurrence (LRR) and distant metastasis (DM). LRR was defined as persistent disease or any recurrence in cervix, uterus, vagina, adjacent pelvic structures, or regional lymph nodes including pelvic or para-aortic LNs. DM was defined as recurrence occurred in non-regional LNs or visceral metastases.

### Statistics

Disease-free survival (DFS), loco-regional recurrence free survival (LRRFS), distant metastasis free survival (DMFS), and overall survival (OS) was defined from the time of diagnosis to the time of first evidence of relapse or death from any cause. Patients without documented evidence of recurrence were censored at the date of last follow up visit. Cumulative survival rate was calculated with the Kaplan–Meier method using SPSS ver. 17.0 (SPSS Inc., Chicago, IL, USA). Toxicities are reported as counts with percentages.

## Results

### Patients characteristics

Between July 2010 and January 2013, 52 eligible patients were enrolled. The first patient was enrolled on July 11th, 2010 and the last patient was enroll on January 26th, 2013, respectively. Participants were mainly recruited by direct discussion with potentially eligible subjects who were transferred to our center about the potential benefit and disadvantage of this trial. Besides, recruitment advertisement or poster were also used for participants recruitment. Patient characteristics are summarized in Table 1. Median age of these patients was 55 years (range: 39–67 years). The number of patients with stage IIB, IIIB, and IVA disease was 16, 34, and 2, respectively. Thirty-nine patients had radiologically enlarged pelvic lymph nodes (PLNs) evaluated to be metastatic, among which 12 had 1 positive node, and the remaining 27 patients had 2 or more radiologically enlarged pelvic lymph nodes. Ten out of the 39 patients with positive PLNs had synchronous para-aortic lymph nodes (PALNs) metastasis. The median shortest diameter of enlarged nodes was 1.2 cm (range: 1.0–3.7 cm).

**Table 1:** Patients characteristics

Characteristics	values
Median age (range)-years	55 (39 ~ 67)
ECOG	
0	43
1	9
International FIGO Stage-No. (%)	
IIB	16
IIIA	0
IIIB	34
IVA	2
Tumor Grade-No. (%)	
Well-differentiated	5
Moderately-differentiated	29
Poorly-differentiated	18
Median diameter of primary tumor (range)-cm	5 (3–7)
Pelvic lymph nodes involvement -No. (%)	
positive	39
negative	13
Paraaortic lymph nodes involvement -No. (%)	
positive	10
negative	42
Median of the shortest diameter of LNs (range)-cm	1.2 (1.0–3.7)
Median cumulative point A EQD2 doses (range)-Gy	
IIB	93.8 (83.9 ~ 93.8)
IIIB ~ IVA	99.6 (93.8 ~ 109.5)
Median duration of OTT of radiotherapy-days	46.5 (42–66)
Median cycles of concurrent chemotherapy	5 (1–6)
Median cycles of adjuvant chemotherapy	2 (1–3)

### Radiotherapy

All the 52 patients completed intended radiation schedule. A median of 50 Gy was prescribed to the PTV (range, 45-50 Gy), whereas PTV-nodes were boosted to a median of 60 Gy (range, 50.0-65 Gy). EF-IMRT was administered in 22 patients, including 10 with positive PALNs and 12 treated prophylactically. In IMRT, the mean volume receiving 40 Gy or more (V40) for rectum, bladder, and intestine was 40.4 ± 15.7%, 38.7 ± 10.2%, and 23.2 ± 6.9%, respectively, and the mean volume receiving 50 Gy or more (V50) for rectum and bladder was 15 ± 7.7% and 12.8 ± 5.4%, respectively.

A median of 35 Gy (range, 28-42 Gy) of brachytherapy boost were prescribed to point A. The median bladder at ICRU 38 reference points from brachytherapy was 21.6 Gy (range, 18.5 Gy-26.46 Gy). The median rectum doses at ICRU 38 reference points from brachytherapy was 18.2 Gy (range, 15.7 Gy—25.6 Gy).

The median cumulative EQD2 doses prescribed to point A was 93.8 Gy (range, 83.9 ~ 93.8 Gy) for patients with stage IIB disease and 99.6 Gy (range,93.8 ~ 109.5 Gy) for those with IIIB-IVA disease, respectively. The median overall treatment time (OTT), including the duration of EBRT and brachytherapy, was 46.5 days (range, 42–66 days). Radiotherapy interruption exceeding 3 days was documented in 5 patients, among which 3 were related to delayed recovery of myelosuppression and 2 were related to acute gastrointestinal toxicities.

### Chemotherapy

A total of 257 cycles of concurrent cisplatin were administered with a median of 5 cycles (range, 1–6 cycles), and the majority (79%) of patients completed 5–6 cycles. Two patients discontinued concurrent cisplatin within 2 cycles, one related to grade 3 vomiting at first cycle and the other related to grade 3 myelosuppression occurred at 2nd cycle of chemotherapy.

A total of 50 patients received adjuvant chemotherapy, while above mentioned 2 patients who discontinued concurrent chemotherapy declined further medical intervention and withdrew from the trial. Thirty-nine (78%) patients completed planned 2 cycles of adjuvant chemotherapy. Two patients received only 1 cycle due to grade 3 thrombocytopenia. As for the remaining 9 patients, serum squamous cell carcinoma antigen (SCCA) levels did not return to normal until 3rd cycle was administered. Median interval between the completion of CCRT and the initiation of adjuvant chemotherapy was 26 days (range, 23 ~ 35 days).

### Outcomes

Fifty patients achieved complete response (CR) 4 weeks after the completion of the treatment protocol, whereas the remaining 2 had persistent disease. After a median follow-up of 66 months (range, 17–97 months), 42 patients were still alive without disease. Detailed information of treatment failure was listed in Table 2. In brief, loco-regional (including 2 persistent disease), distant, and synchronous treatment failure occurred in 4,5, and 1, respectively. The 5-year DFS, LRRFS, DMFS was 80.5%, 90.3%, and 88.0%, respectively. As for the 39 patients with positive nodes, 1 patient with 1 single positive PLN had out-field para-aortic metastasis, 1 patient with multiple PLNs was with persistent disease, 3 patients with enlarged para-aortic nodes developed distant LNM including mediastinum or supraclavicular LNMs, and 2 patients with enlarged para-aortic nodes developed distant hematologic spread including lung and/or liver metastasis. The remaining 32 patients with positive nodes were alive without disease at last follow-up.

**Table 2:** First site of treatment failure (including persistent disease)

First site of recurrence	No. of patients (%)
Loco-regional recurrence	4
Persistent disease	2
Vagina	1
PALN outside radiation field	1
Distant metastasis	5
Lung	1
Lung and liver	1
Mediastinum or supraclavicular LNs	3
Synchronous LRR and DM	1
Synchronous pelvic side-wall, PLN, PALN, and supraclavicular LNs	1

**Fig. 2:**
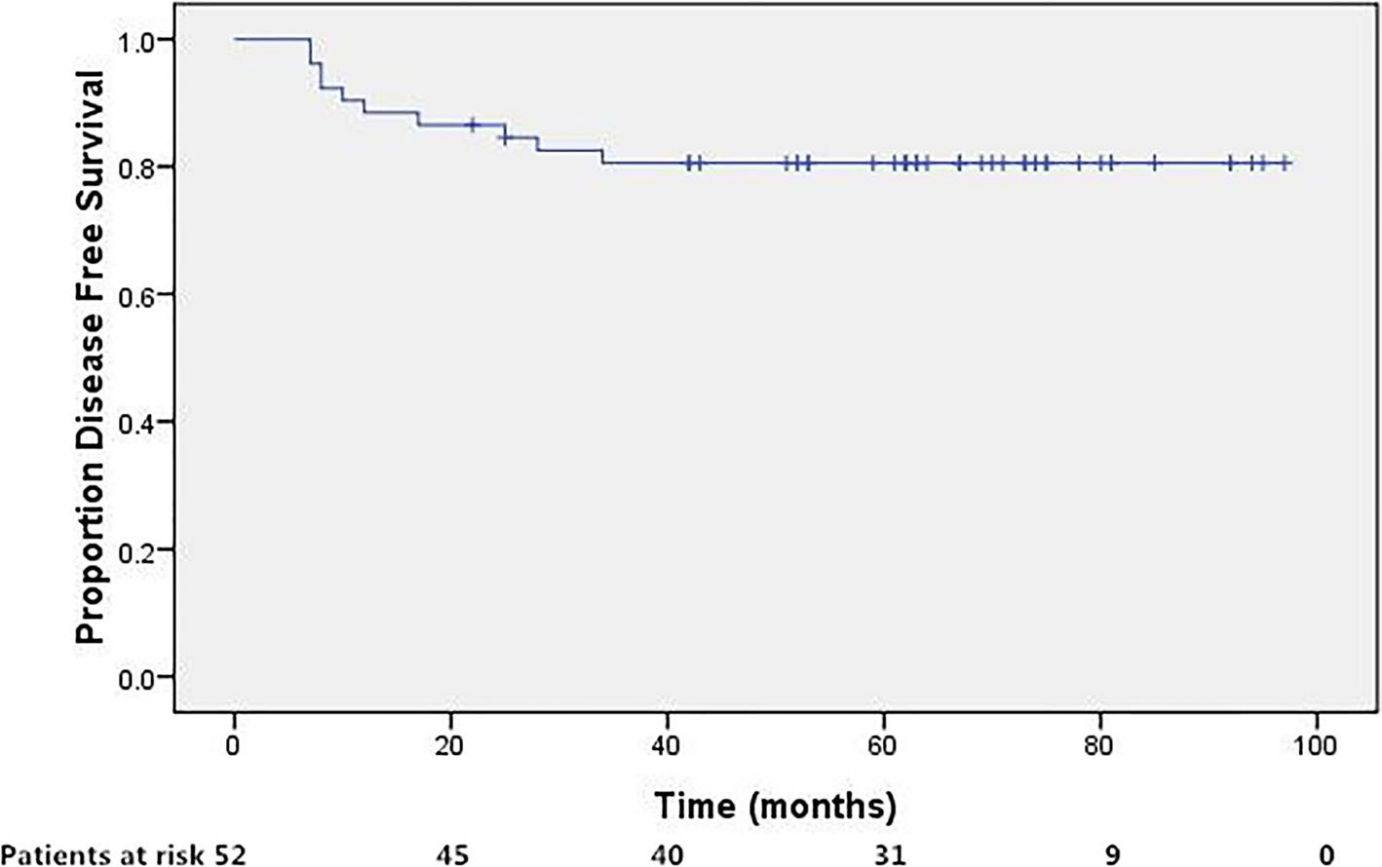
Disease free survival for patients treated with concurrent CCRT incorporation IMRT followed by adjuvant TP regimen. The 5-year disease-free survival was 80.5%

**Fig. 3:**
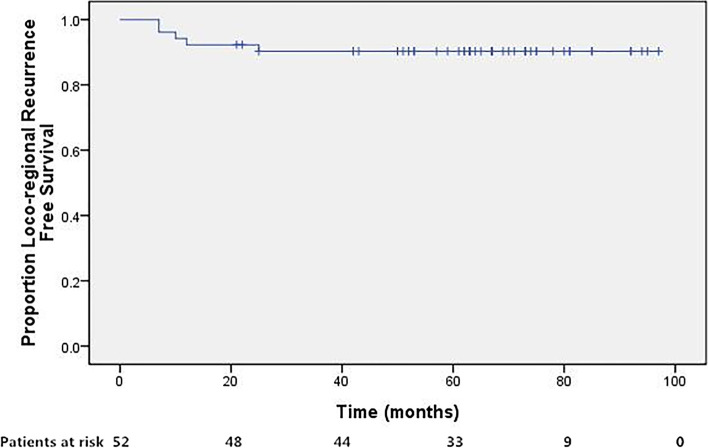
Loco-regional recurrence free survival for patients treated with concurrent CCRT incorporation IMRT followed by adjuvant TP regimen. The 5-year loco-regional recurrence-free survival was 90.3%

**Fig. 4:**
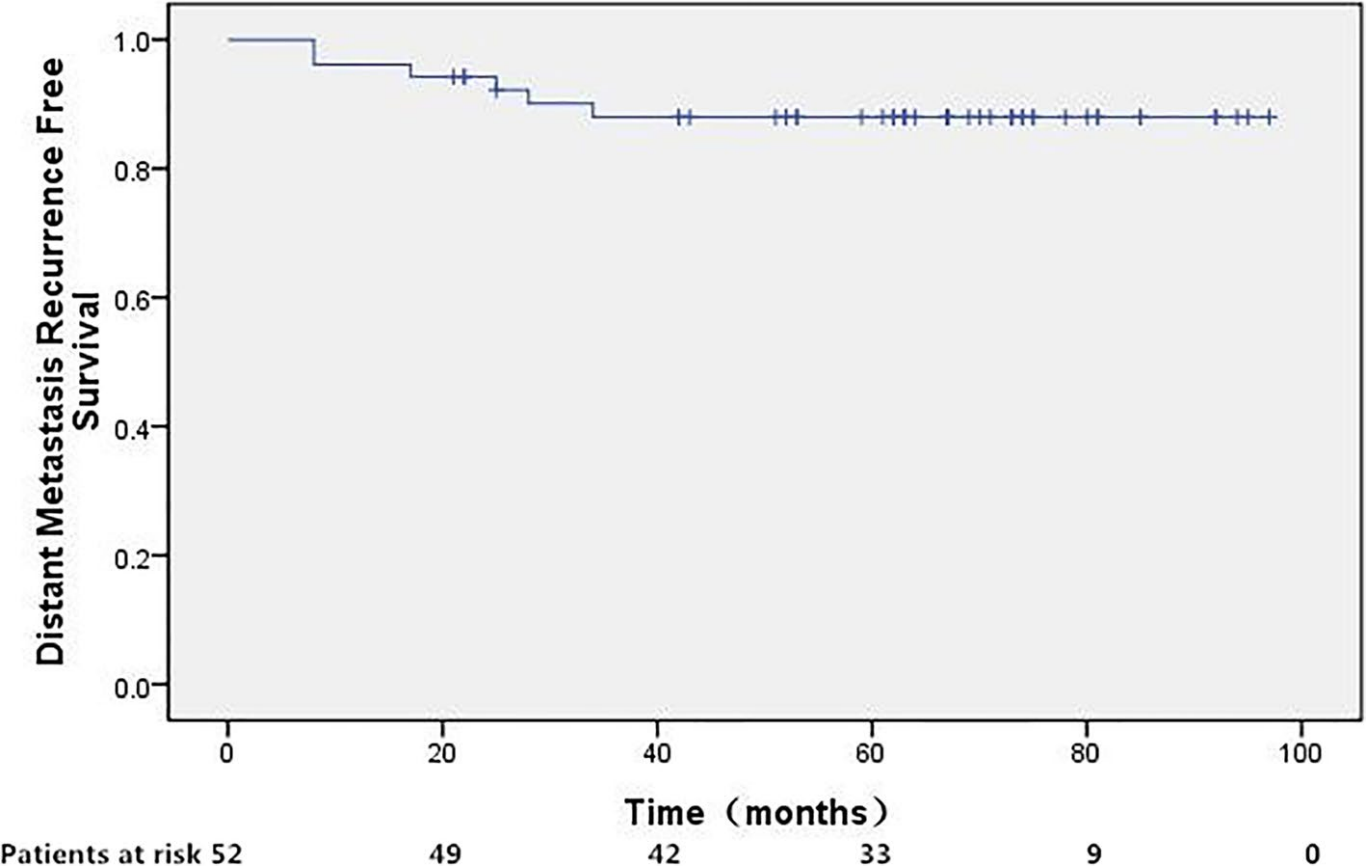
Distant metastasis free survival for patients treated with concurrent CCRT incorporation IMRT followed by adjuvant TP regimen. The 5-year distant-metastasis free survival was 88.0%

**Fig. 5:**
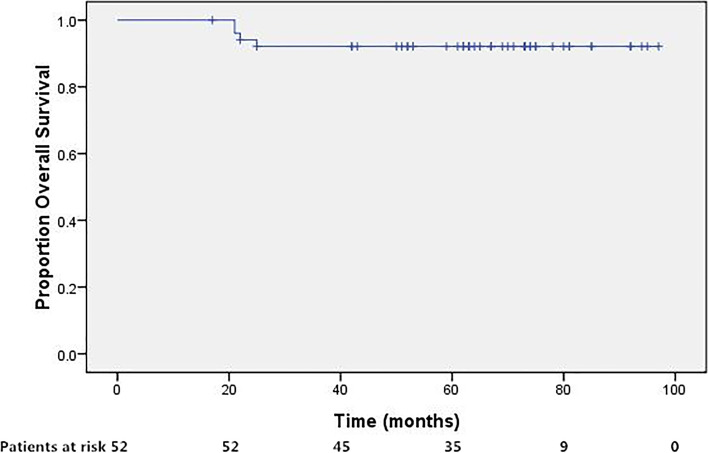
Overall survival for patients treated with concurrent CCRT incorporation IMRT followed by adjuvant TP regimen. The 5-year overall survival was 92.1%

Four of the patients succumbed to the disease, including 2 with persistent disease, 1 with hematological relapse, and 1 with synchronous relapse. The remaining patients were successfully salvaged, as 1 vaginal relapse salvaged by surgery, 1 pulmonary metastasis by chemotherapy, and all the 4 lympho-genous recurrence occurred in radiation-naïve nodes and were salvaged by radiotherapy. The 5-year OS was 92.1%. The survival curves were shown in Figs. 2, 3, 4, 5.

### Toxicities

The incidence and categories of toxicities are summarized in Tables 3, 4. In brief, gastrointestinal and hematological toxicities were mostly reported acute complications during CCRT, which were usually mild and transient. The occurrence of hematological toxicities elevated mildly during adjuvant chemotherapy, as 32% (16/50) and 4% (2/50) patients experienced grade 3–4 leukopenia and thrombocytopenia, respectively. Yet, none of the patients developed febrile episodes or required blood transfusion. Delayed chemotherapy schedules and reduced dosage was required in 14 patients (28%) and 4 patients (7.7%), respectively. Grade 3–4 late toxicities were reported in 3 patients, including 1 vesicovaginal fistula (grade 4), 1 recto-vaginal fistula (grade 4), and 1 intestine obstruction requiring surgical intervention (grade 3).

**Table 3:** Acute toxicity of CCRT and adjuvant chemotherapy

Toxicities	CCRT (*n* = 52)					Adjuvant chemotherapy (*n* = 50)				
	Grade0	Grade1	Grade2	Grade3	Grade4	Grade0	Grade1	Grade2	Grade3	Grade4
Hematologic toxicity										
Neutropenia	8(15.4%)	9(17.3%)	29(55.8%)	6(11.5%)	0(0%)	6(12.0%)	8(16.0%)	20(40.0%)	15(30.0%)	1(2.0%)
Febrile neutropenia	52(100%)	0(0%)	0(0%)	0(0%)	0(0%)	50(100.0%)	0(0%)	0(0%)	0(0%)	0(0%)
Anemia	27(51.9%)	13(25.0%)	12(23.1%)	0(0%)	0(0%)	20(40.0%)	6(12.0%)	24(48.0%)	0(0%)	0(0%)
Thrombocytopenia	49(94.2%)	2(3.8%)	1(1.9%)	0(0%)	0(0%)	33(66.0%)	11(22.0%)	4(8.0%)	2(4.0%)	0(0%)
Non-hematologic toxicity										
Nausea/vomiting	0(0%)	34(65.4%)	14(26.9%)	4(7.7%)	0(0%)	0(0%)	36(72.0%)	12(24.0%)	2(4.0%)	0(0%)
Diarrhea	35(67.3%)	11(21.2%)	4(7.7%)	2(3.8%)	0(0%)	45(90.0%)	3(6.0%)	2(4.0%)	0(0%)	0(0%)
Bladder toxicity	44(84.6%)	6(11.5%)	2(3.8%)	0(0%)	0(0%)	50(100%)	0(0%)	0(0%)	0(0%)	0(0%)
Hepatologic	50(96.2%)	2(3.8%)	0(0%)	0(0%)	0(0%)	47(94.0%)	2(4.0%)	1(2.0%)	0(0%)	0(0%)
Renal	51(98.1%)	1(1.9%)	0(0%)	0(0%)	0(0%)	49(98.0%)	1(2.0%)	0(0%)	0(0%)	0(0%)

**Table 4:** Late treatment-related toxicities

	No. of patients				
Toxicities	Grade0	Grade1	Grade2	Grade3	Grade4
Gastrointestinal	19	24	7	1	1
Genitourinary	36	13	2	0	1

## Discussion

### Principal findings

As far as we know, the current study represents the first publication investigating the efficacy and safety of incorporating IMRT and adjuvant TP regimen into the treatment of high risk LACC patients. We found that such a paradigm was associated with a markedly improved survival with well-tolerated side effects, as estimated 5-year OS, DFS, LRRFS, and DMFS was 92.1%, 80.5%, 90.3%, and 88.0%, respectively, which compared favorably with most previous reports composed of similar study population.

In the early years, a median of summed EQD2 dose of 77.1 Gy had been used, which was soon proved to be insufficient for bulky tumors [23]. In a recent study, Meng et al. revealed that higher EQD2 dosage to Point A (≥ 98 Gy) was associated a significant LRRFS advantage [24]. In our series, a median EQD2 of 93.8 Gy for stage IIB-IIIA patients and 99.6 Gy for stage IIIB-IVA yielded a complete response of 96.2%, reiterating that higher radiation dosage prescribed to point A still constitutes the cornerstone for the definitive treatment of LACC even in the era of modulated radiotherapy.

It has been reported that approximately 40% patients with adenopathy were not able to obtain a complete nodal response if treated with conventional radiotherapy, and 46.2% of them would recur at 2 years [25]. By contrast, nearly 90%-100% involved nodes resolved completely when IMRT boost was used, and only 0%-8% of them relapsed [23, 26–29]. Consistently promising results were reported in the current study when a median IMRT boost of 60 Gy was prescribed. All the involved nodes remised completely, and only one in-field regional relapse occurred synchronously with pelvic and distant recurrence. These findings suggested that IMRT boost was highly effective in nodal sterilization; it should be considered as an indispensable component of treatment for LACC, especially when positive nodes were present.

Except for escalated radiation dosage, SIB technique might be another underlying reason for improved loco-regional control, as it allowed for a maximum of 65 Gy EBRT to be delivered at 2.6 Gy per fraction within a median OTT of 46.5 days [30]. In Guckenberger’ study, increased dose per fraction and reduced OTT through SIB allowed for an iso-toxic dose escalation of 8.0 Gy on average, which consequently yielded an improved tumor control probability from 15 to 28% [31]. It had been recommended that radiotherapy should be completed within 56 days so that potential accelerated cancer cell repopulation could be avoided [32–34]. In an undergoing multicenter study aiming to benchmark a high level of disease control, SIB was regarded as an evolution over the past decades and required in all the participants with pathologic nodes [35]. Its beneficial effect is expected to be further verified.

Occult paraaortic metastases might occur in approximately10 ~ 20% patients with apparently negative PALNs [36, 37]. Yet, Lee et al. revealed that it was in patients with common iliac or > 3 pelvic LNs involvements that EF-IMRT was associated with a superior PALNs recurrence free survival over standard pelvic radiation (100% vs. 56.8%). By contrast, no significant survival difference was observed (100% vs. 93.8%) in patients without these features [29]. Based on these findings, the authors postulated that a risk-guided EF-IMRT seem to be more reasonable. In the present study, EF-IMRT was reserved for patients with involved common iliac nodes or bulky PLNs. Our reported 5-year PALNs recurrence free survival of 97.9% further validated the highly effectiveness of such a risk-guided policy in the management of paraaortic lymph-node metastasis.

The value of adjuvant chemotherapy might had been underestimated in previous literatures when stage I-II patients were enrolled [15, 16]. It was in a study made up of 48.7% stage III-IV patients and 70% patients with LNM that a discernable DMFS advantage (20.5% vs. 30.8%) in favor of consolidating cisplatin plus 5-fluorouracil regimen was observed [17]. Further improved outcomes were reported when more potent cytotoxic combinations were used. In Zhang’s paper, adjuvant paclitaxel plus nedaplatin (TN) regimen was associated with a 3% occurrence of DM in a cohort containing 70% stage III patients [21]. In 2021, the results of OUTBACK Trial, a randomized phase III comparing the treatment outcome of adjuvant chemotherapy following chemoradiation or chemoradiation alone in the treatment of primary treatment for locally advanced cervical cancer, was reported [38]. A total of 919 patients were enrolled, and no survival benefits were disclosed between the 2 arms either in terms of overall survival (72% vs. *P* = 0.91) or disease-free survival (63% vs. 61%, *P* = 0.61). Yet, over 3/4 enrolled patients were with stage I-II disease, and 23% of the population fail to complete chemoradiation. Similarly, no significant benefit of paclitaxel with carboplatin given for 3 cycles after a standard concurrent chemoradiation treatment for LACC was demonstrated in another randomized controlled trial, the ACTLACC study [39]. The authors postulated that the benefit of adjuvant chemotherapy might be diluted by including over 60% stage II patients which might be managed by chemoradiation alone, or may be due to the less effectiveness of paclitaxel plus carboplatin regimen in the treatment of LACC. Therefore, the role of adjuvant chemotherapy following chemoradiation in high risk LACC still remain to be classified. In our current series composed entirely of high-risk patients, adjuvant TP regimen led to a 5-year DMFS of 88.0%, which compared favorably with previously reported data achieved in similar population. These observations indicated that in high-risk LACC patients, adjuvant paclitaxel-platinum combination chemotherapy might play an important role for reduced distant metastasis.

Several other first-line regimens for recurrent of metastatic cervical cancer had also been evaluated in the adjuvant setting with encouraging oncologic outcomes, including gemcitabine plus cisplatin (GP) or paclitaxel plus carboplatin (TC) regimen, etc. [18, 19]. However, nearly 90% of the participants prescribed with GP regimen experienced grade 3–4 complications including 2 possibly toxicity-related deaths, whereas TC -based CCRT followed by adjuvant chemotherapy was associated with a 60.0% occurrence of grade 3–4 acute hematological toxicity or even higher [18, 20]. In comparison, TP or TN regimen seem to be more tolerable, as severe hematological toxicities were reported to be 36% and 21.8% in the present study and in Zhang’s study, respectively [21]. In this regard, TP or TN regimens seemed to be preferred adjuvant options following CCRT.

Yet, Choi et al. revealed that although hematological relapse was statistically reduced by adjuvant PF regimen, the occurrence of non-regional lymphogenous relapse remained unaffected [17]. In a series consisted entirely of patients with lymphadenopathy, Abe et al. even failed to disclose an improved DFS when adjuvant TC regimen was administered [20]. A similar trend was observed in the current study, as hematological spread was diminished to the low of 3.8%, while out-field lymphogenous metastasis occurred in 9.6% of the patients. Therefore, further investigations should be focused on exploring novel agents more effective in sterilizing tumor cells harbored in lymph nodes.

Due to the relatively small study population and lack of valid control group, a selection bias could not be avoided in the current study. Another major limit of the current study lies in the unbalanced treatment combination of high-precision EBRT and two-dimensional brachytherapy, as this study was initiated over 10 years ago when IGRBT was not widely available. Due to the 2D brachytherapy used, OARs including rectum and bladder doses in brachytherapy could not be accurately measured, although 3 grade 3 ~ 4 late GI and GU toxicities occurred. Nevertheless, the strength of this study lies in that it represents the largest study composed homogeneously of high-risk patients treated with a well-established highly effective cytotoxic regimen, so that confounding effects of the enrollment of low-risk patients or less potent regimens were minimized.

## Conclusions

This current study demonstrated that IMRT and TP chemotherapy were highly effective and well-tolerated in the treatment of high-risk LACC. By means of ensured target volume coverage, escalated nodal boosts, enhanced biological effects, and tailored radiation fields, IMRT yields an improved loco-regional control. Meanwhile, distant metastases could be effectively eradicated with mild toxicities when adjuvant TP regimen was prescribed. Larger prospective randomized controlled trial studies are warranted to further validate these findings.

## Data Availability

All data generated or analyzed during this study are included in this published article.
